# We need higher education: Voice of nursing administration from Kabul, Afghanistan

**DOI:** 10.1002/nop2.140

**Published:** 2018-03-25

**Authors:** Wais M. Qarani, Rafat Jan, Khwaja M. I. Saeed, Laila Khymani

**Affiliations:** ^1^ French Medical Institute for Children (FMIC) Kabul Afghanistan; ^2^ School of Nursing and Midwifery Aga Khan University School of Nursing and Midwifery (AKU‐SoNaM) Karachi Pakistan; ^3^ Head of Grant and Contract Management Unit (GCMU) Ministry of Public Health Kabul Afghanistan; ^4^ Aga Khan University Hospital (AKUH) Karachi Pakistan

**Keywords:** Afghanistan, continuous professional development, higher education, nursing administration, public hospitals

## Abstract

**Aim:**

To explore the educational profile of nursing managers and head nurses at public hospitals in Kabul, Afghanistan.

**Design:**

A descriptive cross‐sectional study design was employed.

**Method:**

A self‐administered pre‐tested questionnaire was used to recruit 86 nursing managers and head nurses from 17 public hospitals in Kabul. SPSS version 19 was used to analyze and report the data through descriptive statistics.

**Results:**

It was found that, none of the participant was prepared with higher education in nursing; rather they had only diploma in nursing; and 84.9% of them had completed their nursing diploma before 2002.; 11.6% of participants were currently studying; and all were in non‐nursing disciplines. On the other hand 100% of the participants expressed intention for further studies mainly in leadership and management, computer skill, English language, in‐service nursing trainings and higher education in nursing.

## 1 BACKGROUND

In nursing both conceptual and experiential learning are important and both the approaches have critical role in the development of competencies. However, the domain of conceptual learning will be a mediator to excel in any other domain of professional development. An effective nurse manager with leadership skills and abilities to positively influence the work environment will stimulate greater achievement and enhance organizational outcome (McGuire & Kennerly, 2006). The efficiency of leaders and managers is dependent on the level of their education; and those who have received advanced education are more efficient (Elmuti, Minnis, & Abebe, 2005). Similarly, a study found that higher education in nursing has played a significant role in the development of nursing profession in Pakistan (Gul, Paul, & Olson, 2009). Various international researches link BScN‐prepared nurses to improved patient outcome (Aiken, 2003; Cheung & Aiken, 2006; Megginson, 2008; Yakusheva, Lindrooth, & Weiss, 2014) and cost effective care (Yakusheva et al., 2014). Thus, appropriate educational preparation is necessary for an advanced nursing practice; and enables nurses to quantify their contribution in health system (Furlong & Smith, 2005). Adequate educational preparation is essential for the nursing leaders to translate key strategies into action. Therefore, nursing leaders have to be adequately educated and trained to execute strategies to address deficiencies in nursing education and nursing practice.

The Afghan constitution recommends free education till the Bachelors level to the population (Afghan Const. art. 43, Chapt. 2). Basic nursing education in Afghanistan is still at the diploma level and is offered by both public and private sectors. Basic nursing education in the public sector is provided by nine Institutes of Health Sciences (IHSs) throughout the country. These institutes, which prepare diploma nurses, are centralized and managed by the Ministry of Public Health (MoPH). Although IHSs are managed by the MoPH, the students get admission to these institutes through the national examination system, known as Conquer Exam, through the Ministry of Higher Education (MoHE). There are pre‐defined criteria of the Conquer Exam that decide students' admission to any discipline. A drawback of this system is that those students who are unable to fulfil the requirements of Bachelors programs are directed to the nursing profession and other allied health programs. Since the students' admission to the nursing program is based on the result of Conquer Exam rather than students' choice; students may not have interest and may not perform adequate during their academic progression.

Moreover, a baseline assessment of the Kabul Institute of Health Sciences (IHS), in 2002, revealed that, the administrators had not had any exposure to the standardized and current methods of educational administration. The teachers of IHS were not computer literate, had little ability to comprehend the English language and were not exposed to the international standards of nursing education (Herberg, 2005). However, during the last decade there have been improvements in the development of policy documents and regulation in nursing education. Revised curricula, faculty development (Herberg, 2005), national policy on accreditation of nursing schools and recognized nursing education standards (AMNEAB, 2011), national policy and strategy for nursing services and in‐service nursing standards (Nursing and Midwifery Department (NMD), 2011) are significant progresses that have taken place in nursing in Afghanistan during the last decade. However, little attention is given in national policy and strategy to develop the competencies of nursing leaders to effectively execute the mentioned key developments.

Despite that, nurses with higher education perform more competently in leadership and management positions compared with those who are not adequately trained and not prepared through higher education; surprisingly, in Afghanistan as of 2017 there is only one nursing program in the entire country, which prepares baccalaureate nurses. The program with a standardized and comprehensive curriculum was initiated in 2006 (KMU 2013) in the Kabul Medical University (KMU) and is managed by the Ministry of Higher Education. The latest establishment and being the only BScN program in Afghanistan explains the fact that nursing leaders may not fulfil the requirement, i.e., a Bachelors degree, for them to hold leadership and managerial positions in the nursing profession. The educational profile which refers to the basic and professional education; and continuous professional development trainings for the nurses is pivotal for organizational outcome. Therefore, this study aimed to determine the educational profile of Nursing Managers (NMs) and Head Nurses (HNs) at public hospitals in Kabul, Afghanistan. Subsequently, the findings of this study will inform policy makers at MoPH to plan appropriate strategies for developing the capacity of the nursing leaders; and to ensure patient safety and enhance outcome through an effective leadership.

## 2 METHODOLOGY

### 2.1 Study design

A descriptive cross‐sectional study design was used to determine the academic profile of the nursing leaders at public hospitals in Kabul, Afghanistan.

### 2.2 Study setting

The setting for this study was all 17 public hospitals in Kabul, which are directed by the Directorate Central Hospitals, General Directorate Curative Medicine, MoPH. Other public hospitals under different ministries and private sector hospitals were excluded from the study due to the time constraints and limitation of resources.

### 2.3 Study sample and sampling

Total population sampling technique was used purposively; and all NMs and HNs who were nurses by the profession and were on duty during the data collection were approached to participate in the study. The data were collected while the PI was present on the data collection site, assisted by a research assistant, both being accessible in the field to clarify any query. Participation was voluntary and enough time was given to the participants to complete the questionnaire. Total 100 eligible participants were accessed and the response rate was 89% (89 participants). Finally, three incomplete forms were also excluded from the analysis plan, which brought the actual sample size down to 86 participants. In this study, NM refers to a nurse leader responsible for directly supervising the HNs and managing multiple units of a hospital, such as inpatient and outpatient departments. HN refers to is a nurse leader who reports directly to the NM and has the responsibility of managing a single unit in a hospital. The HN is responsible for the day to day operations and supervision of nursing personnel.

### 2.4 Data collection

The data were collected during January–February 2015 using a self‐administered questionnaire. In this paper, a section of the main questionnaire which was developed for a thesis conducted in partial fulfilment for the Master of Science in Nursing degree is reported. This section of the questionnaire contained questions on: a) educational characteristics of the participants in six categories; and b) Training needs of the participants in three categories. Although categories were defined for the training needs; an option was also given if they wish to add any other training needs. Content validity of the questionnaire was carried by a panel of experts. The questionnaire was translated into the Dari language and then back translated into English, without any major differences or loss of meaning after back translation. Moreover, the questionnaire was pre‐tested on 11 participants with the aim to validate the Dari translation, identify areas that are culturally sensitive and to identify limitations in the questions. The final tool was administered for actual data collection after incorporating relevant modifications.

### 2.5 Ethical consideration

The Institutional Review Board (IRB) of the Ministry of Public Health approval (479716/23‐Aug‐2014) and Ethical Review Committee (ERC) of the Aga Khan University approval (3349‐SON‐ERC‐14/05‐Jan‐2015) were granted before conducting the study. The approval to access the study settings i.e., 17 hospitals, was also taken from the Directorate Central Hospital, General Directorate Curative Medicine, MoPH. Informed consent was taken from each participant before the data collection. Institutional affiliation and identifiable demographic information of the participants were coded, to ensure anonymity and confidentiality.

### 2.6 Data analysis

The principal investigator was available on‐site to verify that the questionnaire is filled completely and correctly to ensure quality data collection. The collected data were entered into Epi Info‐7, to avoid any error during the data entry, after which they were imported to Statistical Package for Social Sciences (SPSS) version 19, for analysis. Descriptive statistics were used to analyse this section of the study and the results were presented in frequencies and percentages.

## 3 RESULTS

There were 14 NMs and 72 HNs in this study. The educational characteristics of the respondents varied considerably (see Table 1). More than one‐fourth of the participants (25.6%) had completed the basic school education till grade‐IX only (9 years of basic school education, starts when the child reaches eight years of age; also known as secondary school graduation), before enrolment into professional education. The duration of professional education was 2 years for 14% and 3 years for 86% of the participants. Majority of the participants (69.7%) held a diploma in nursing and 14% had diploma in nursing and midwifery, whereas 16.3% had Feldsher, which is a 3 year phased‐out diploma program equivalent to 3 year diploma nursing. A significant proportion of the participants (84.9%) had completed the professional education before the year 2002, after which considerable improvements in nursing education including revision of national nursing curriculum had initiated. Only 15 (17.4%) participants received some sort of certified in‐service training and the duration of the training ranged from 1–12 months.

**Table 1:** Educational characteristics of the study participants

Variables	*N*	Frequency	Percentage
Basic education	86		
Grade IX		22	25.6
Grade XII		64	74.4
Professional education	86		
Diploma in nursing		60	69.7
Diploma in nursing and midwifery		12	14.0
Feldsher		14	16.3
Duration of professional education	86		
2 years		12	14.0
3 years		74	86.0
Year of graduation from professional education	86		
Before 2002		73	84.9
After 2002		13	15.1
In‐service managerial training taken	86		
Yes		15	17.4
No		71	82.6
Duration of in‐service managerial training in months	14^a^		
1–6		13	98.8
7–12		01	01.2

a Out of 15 participants who have taken in‐service managerial training, one participant did not mark the duration of the training on the questionnaire.

The intention of the participants about attending training was explored, where all of them (100%) expressed an interest for further trainings. Majority of them i.e., 83.7% showed interest in computer training, followed by 81.4% in English language, 79.1% in leadership and management and only 9.3% and 4.65% in in‐service nursing trainings and higher education in nursing respectively (Table 2). It is also worth to mention that, 11.6% of the participants switched from the profession and were pursuing degrees in non‐nursing disciplines mainly; medical, pharmacy and stomatology.

**Table 2:** Training needs of the study participants

Variables	*N* a	Frequency	Percentage
Intention for training	86		
Yes		86	100
No		00	000
Prioritized training needs			
Computer training	86	72	83.7
English language training	86	70	81.4
Leadership and management	86	68	79.1
In‐service nursing training^b^	86	08	09.3
Higher education in nursing	86	04	04.7
Participants studying in non‐nursing disciplines	86		
Yes		10	11.6
No		76	88.4

a the letter “*N*” refers to the total number of participants who have answered the question.

b In‐service nursing training topics include: Nursing standards, capacity development, nursing ethics, and project management.

## 4 DISCUSSION

In several countries of the world, many hospitals have started offering nursing positions to only those nurses who have a Bachelor degree (Kelly‐Williams, 2011). However, in our study, none of the NMs and HNs had acquired Bachelor degree in nursing education and one‐fourth of them had not even completed 12 years of basic school education before enrolling into a two‐year or three‐year nursing education program. There are limited studies on nursing administration from the neighbouring countries to compare our findings. However, there are numerous studies from other countries which show a huge gap. For instance, 24.8% of the nursing administrators in Taiwan had masters degree (Kang et al., 2012) and 36.2% of the nursing managers in South Africa had degree in nursing (Pillay, 2011). Furthermore, it is reported by Opiyo and English (2010), that improved performance was associated with training. However, more than 80% of the participants in the current study had not received any in‐service training as part of the continuous professional development (CPD) during the last 2 years. These findings illustrate that the academic profile of the nursing leaders in this setting is not adequate. Therefore, the findings in regard to the intention of the participants to receive training in computer skills, English language and leadership and management strategies is significant for the policy makers to design capacity development strategies for the nursing leaders.

In the second section of the questionnaire which was about determining the training needs of the participants (Table 2), it was recognized that all participants of the study expressed intention for further training. Almost 80% of the participants expressed interest for all enlisted categories of trainings which were: 1) computer skills literacy training; 2) English language proficiency training, and 3) leadership and management trainings.

Considering the new developments and advances in technology; nurses particularly nursing leaders of today must be computer literate. Computer skills are essential, as day to day operational activities and communication are very much dependent on the computer technology. However, in public sector majority of the hospitals still rely on traditional paper work; therefore, more than 83% of the participants expressed their need for computer training. Computer literacy is essential for nurses (Smedley, 2005) and can be availed for different purposes. Computer technology facilitates to share knowledge and provide services through Tele‐conference even to the most remote areas of the country. Furthermore, computer literacy facilitates to view the world and discover new innovations and evidence‐based practices in the health care around the world. But, to view the world, there is a need to understand the global language which is English beside local languages. Afghanistan is in the initial phase of struggling for development; where majority of the systems are yet need to be improved. Education in schools and universities are in local languages; and there is a limited opportunity for the students in public sector to learn English language. Therefore, more than 80% of the participants in our study expressed that they need English language training, which will enable them to comprehend discoveries around the world especially advanced and up‐to‐date learning and education even in leadership and management concepts. For this, the ministry of public health may design strategies to fulfil the training needs of the nursing administration in these areas.

In addition, another open option was also provided in the questionnaire for the participants, so they may enlist their need for trainings if the defined categories do not capture their need. Surprisingly, 4.7% of the participants highlighted higher education in nursing as an important area for their career development. This percentage is as low and it may be due to the fact that the option of higher education was not subjectively mentioned along with other categories in the questionnaire. Moreover, the participants also highlighted in‐service nursing training such as nursing standards, capacity development, nursing ethics and project management as their training needs.

The study also revealed that only 11.6% of the participants were currently studying. But, their areas of study were all in non‐nursing disciplines and this might be due to the fact that there is only one BScN program for the entire country which does not fulfill the need. Although, no study was found to compare the turnover of the nursing administration from the profession; a study of ten European countries indicated that 9% of the nurses intended to leave the profession (Heinen et al., 2013). On the other hand it is found by Nogueras (2006) in her study that: “the higher the RN level of education, the greater the RN occupational commitment and the lower the RN intent to leave the nursing profession” (p. 91). Thus, the finding from the current study and unavailability of BScN programs in the country makes a case at policy level to upgrade nurses' educational profile through CPD and higher education.

The experience of Pakistan in implementing the Post Registered Nurse Bachelors of Science in Nursing (Post‐RN BScN) program may be replicated in Afghanistan to fulfil the need for higher education (Upvall, Karmaliani, Pirani, Gul, & Khalid, 2005). Post‐RN BScN is a Two‐year nursing education which upgrades diploma‐prepared nurses to bachelorette‐prepared nurses.

### 4.1 Implication for nursing and health policy

The global standards for the initial professional nursing education call that all nurses to be prepared with bachelor's degree (WHO 2009), which is important for patient safely. On the other hand, the current study determined the actual picture of the nursing managers' and head nurses' educational preparedness who lead in the healthcare settings in Kabul.

The gap found from the current study on the compliance with the global standards urge policy makers in the Ministry of Public Health to design policy and strategies for the paradigm shift from traditional diploma nursing to degree nursing programs in the country. Bachelorette prepared nurses are important for an effective and efficient leadership; and also will play an important role in the provision of safe and quality care to the patients, which ultimately will have its impact on the rate of morbidity and mortality in the healthcare settings.

### 4.2 Strengths and limitation of the study

The major strength of this study is that, to the best of the PI's knowledge, it is the first nursing study in Afghanistan that identified the educational profile and training need of the NMs and HNs in public sector in Kabul. Although the findings of this study cannot be generalized to the entire population, the study collected rich information on the phenomena and this suggests further studies in nursing in the country. The study also had limitations mainly; some of the participants who were on leave were not included in the study; while their participation might have changed the study findings. The study questionnaire did not capture context relevant variables such as higher education.

### 4.3 Recommendations

First, to develop the competency of NMs and HNs in the public hospitals, the current study suggests two types of capacity development and training programs:

- The first recommendation is higher education in nursing to upgrade the current diploma nurses into degree graduates (Post‐RN BScN), as it is a pressing need of the nurses in the country.
- The second recommendation on in‐service or specialization trainings, such as leadership and management, computer literacy and English language, with the aim to build their capacity or refresh their knowledge is essential to enable them to compete in the evolving market. Graduate studies for the existing NMs and HNs may require extensive planning and plenty of resources; however, MoPH needs to pay more attention towards in‐service managerial short courses to develop the capacity of the existing management workforce of the public hospitals.

Second, a qualitative study could be conducted to explore the reasons why nurses contemplate leaving this profession.

## 5 CONCLUSION

This study suggests that there is a dire need to design both short and long‐term strategies for the capacity development of nursing leaders at public hospitals in Kabul, Afghanistan.

## CONFLICT OF INTEREST

No conflict of interest has been declared by the authors.

## AUTHOR CONTRIBUTIONS

WQ, RJ, Study design; WQ, Data collection; WQ, RJ, KS, Data analysis; Study supervision: RJ WQ, Manuscript writing; WQ, RJ, KS, LK, Critical revisions for important intellectual content.
